# Restoring nature, enhancing active mobility: The role of street greenery in the EU’s 2024 restoration law

**DOI:** 10.1007/s13280-025-02178-w

**Published:** 2025-04-10

**Authors:** Silviya Korpilo, Elias Willberg, Kerli Müürisepp, Robert Klein, Rory Taylor, Jussi Torkko, Kamyar Hasanzadeh, Tuuli Toivonen

**Affiliations:** 1 Digital Geography Lab, Department of Geosciences and Geography, University of Helsinki, PL 64 (Gustaf Hällströmin katu 2), 00014 Helsinki, Finland; 2 Helsinki Institute of Sustainability Science, University of Helsinki, Helsinki, Finland; 3 Helsinki Institute of Urban and Regional Studies, University of Helsinki, Helsinki, Finland

**Keywords:** Active mobility, Environmental exposure, Nature restoration, Street greenery, Urban greenspace planning

## Abstract

This article argues for the importance of integrating a mobility perspective into urban greenspace planning and practice related to the 2024 EU Nature Restoration Law. Street greenery can play an important multifunctional role in promoting ecosystem services and functions, sustainable mobility, and human health and well-being. However, planners need more evidence on how street vegetation affects health and well-being during everyday active mobility, as well as what type, where and for whom to enhance vegetation. We discuss current advancements and gaps in literature related to these topics, and identify key research priorities to support restoration policy and practice. These include: moving beyond dominant scientific thinking of being in place to moving through space in understanding greenery exposure and experience; use of multiple exposure metrics with attention to temporal dynamics; integration of objective and subjective assessments; and investigating further the role of street greenery in reducing environmental injustices.

## Introduction

Street greening interventions have become increasingly popular in cities’ agendas in order to enhance carbon sequestration, climate regulation, urban liveability and sustainable mobility (i.e. integration of sustainability goals into transport (Holden et al. 2020)). In Europe, this trend became also legally binding in June 2024 when the European Parliament adopted the new Nature Restoration Law. The law sets a target for the EU to restore at least 20% of its land and seas by 2030 including specific measures such as planting three billion trees and increasing urban greenspaces and tree cover canopy cover (Regulation (EU) 2024/1991). Street greenery can play an important multifunctional role in achieving these ecological goals, while also providing various co-benefits for urban residents.

Street greenery encompasses all vegetation along travel routes including street vegetation (e.g. trees, shrubs, flowers) and green spaces (e.g. forests, parks, gardens). It provides various ecosystems services including reducing noise, ambient air pollution, urban heat and stormwater run-off, increasing carbon sequestration and providing habitats for multiple species groups such as birds and pollinators (Coleman et al. 2022; Browning et al. 2024). In addition, street greenery offers key opportunities for citizens to interact with nature while on the move and particularly during active mobility such as walking, cycling or other outdoor modes of travel (Cook et al. 2022). An average EU citizen spends 80 min per day on the move (European Commission 2022) with walking as the most popular way of getting around after car travel. Growing evidence shows that street greenery has various social benefits such as promoting physical activity, relieving momentary stress, fostering social contacts, and contributing to walkable travel environments (Sarkar et al. 2015; Lu et al. 2018; Zhang et al. 2018; Yu and Kwan 2024). Many studies also agree that street greenery can advance sustainable mobility by encouraging the choice of walking and cycling over other transport modes (Tsai et al. 2019; Vich et al. 2019; Wu et al. 2020). In addition, authors have found positive associations between street greenery and travel satisfaction and experience (Snizek et al. 2013; Ta et al. 2021; Song et al. 2022). Greener routes are perceived as more pleasant and quieter (Nawrath et al. 2019), but also safer (Coleman et al. 2021; Zhu et al. 2022), which is one of the most important factors encouraging active mobility.

In this article, we argue for the importance of integrating a mobility perspective into urban greenery planning and practice related to the EU Nature Restoration Law. This can promote not only ecosystem services and functions, but also enhance sustainable transportation and human health and well-being during travel (Poom et al. 2021). However, simply greening cities and their streets does not mean that benefits will follow (Markevych et al. 2017). Planners involved in greening interventions need better understanding on *how* street vegetation affects health and well-being during everyday active mobility, as well as more empirical and context-specific evidence on *what* type, *where* and for *whom* to increase or plant new vegetation. While other scholars have argued for better integration of mobility into sense of place (Gottwald et al. 2024), place attachment (Di Masso et al. 2019) or greenspace exposure research (Kwan 2012; Helbich 2018; Willberg et al. 2024), here we focus on the integration in future restoration and greening practices. We discuss these points in relation to current advances and research gaps in literature, while proposing key priorities for future research. These may serve as entry points for interdisciplinary research, but also as applicable guidance for planners on key aspects to consider in order to maximize co-benefits of green infrastructure along transportation networks.

## The HOW: How street greenery affects health and well-being during active mobility?

The active mobility perspective provides a unique approach to understanding the links between street greenery and human health and well-being. First, it blends spatially-fixed and dynamic conceptual understandings. In this regard, the fixities-flow framework of place attachment in mobility (Di Masso et al. 2019) can present a relevant lens through which street greenery exposure can be understood and planned for. This involves the integration of “fixities” or static, spatially-fixed places of exposure, perceptions, and experience of greenery en route, and “flow”––representing the dynamism of actual movement of people. However, much of how we understand interactions with urban nature still stems from the fixed conceptual understanding of *being in a place*, which has been central to studies using residential neighbourhood or greenspace recreational use perspectives (Keniger et al. 2013; Gascon et al. 2015). For example, extensive cross-country evidence shows that living near, visiting, spending time in, and feeling connected to greenspace, are all positively associated with general and mental health (White et al. 2021; Elliott et al. 2023). Yet, considerable portion of people’s contact with nature is still neglected: namely *moving through space* as part of everyday commute.

Second, an active mobility perspective also requires a rather uncommon approach to studying nature-health interactions because exposure is incidental and mostly unconscious. Incidental exposure occurs when the interaction is an unintended result or a by-product of another activity, such as contact with street trees while cycling to work (Keniger et al. 2013; Beery et al. 2017). Greater clarity is needed on the mechanisms of such unintentional nature interactions and how they mediate psycho-physiological and social benefits. Although current evidence does not provide a clear answer, previous studies have already shown that even indirect contact with nature such as viewing images or viewing nature through a window can improve cognitive performance (Wells 2000; Berman et al. 2008), increase perceived restorativeness (White et al. 2010), and reduce impulsivity (Berry et al. 2015). In addition, while actively moving through space, people experience a series of rapidly changing and momentary environmental stimuli. More longitudinal, intervention and experimental studies (such as randomised controlled trials) are needed to reveal the temporal mechanisms of such momentary exposures and how these may or may not accumulate over time (Frumkin et al. 2017; Markevych et al. 2017).

There are still promising advances in this field. An increasing number of studies have started to address the mobility-related gap in greenspace exposure research by emphasizing the importance of the whole “daily exposome”, i.e. the daily environmental exposures that an individual encounters throughout life, including different activity spaces and people’s everyday travel (Liu et al. 2023). Recent studies have assessed pedestrians and cyclists’ overall exposure to greenery (Willberg et al. 2023, 2024; Khanian et al. 2024), investigated associations between greenery exposure during active mobility and mental health (Roberts and Helbich 2021; Wang et al. 2021), and sought to provide estimations of the total greenspace exposure including both in residential neighbourhoods and during mobility (Wei et al. 2023). However, such studies are still scarce and comprehensive understanding on the health and well-being impacts of greenery during travel remains limited (Poom et al. 2021; Le and Poom 2024). In addition, plurality of metrics, spatial scales and aggregation levels to capture dynamic travel contexts and related street greenery exposure have resulted into mixed results on health and well-being outcomes, requiring more shared and systematic measurement practices (Larkin and Hystad 2019; Yu and Kwan 2024).

## The WHAT: What kind of street greenery is important during active mobility?

First, in order to understand what type and characteristics of street greenery are important for active mobility, there is a need for transdisciplinary research that integrates insights from urban planning and transportation, greenspace exposure, public health, and environmental psychology. Combining objective measures of greenery exposure with subjective assessments of travel experience can help capture individual and perception nuances that can be highly relevant in shaping human well-being and health benefits, as well as overall travel satisfaction (Gascon et al. 2015; Frumkin et al. 2017). However, this is still not a common practice. Greenspace exposure research has focused mostly on objective environmental measures with the exception of a limited number of studies (e.g. Ueberham et al. 2019; Tao et al. 2020; Torkko et al. 2023). Simultaneously, focus on the quantity rather than quality remains prevalent, which limits knowledge on how health impacts can be mediated by biodiversity (e.g. structural diversity, tree richness, pollinator richness) (Fisher et al. 2022) or perceived quality (Korpilo et al. 2024).

In addition, street greenery should be planned as a multisensory environment. People experience multiple simultaneous exposures during everyday travel (Helbich 2018). Research has focused mostly on visual metrics, air pollution and noise, and there are limited studies that include multiple concurrent exposures (Poom et al 2021). This inherently produces bias as considering single exposures can lead to overestimation of individual effects and underestimation of interactions and combined effects (Klompmaker et al. 2019).

Research should also take into account temporal variations more systematically. In fact, one of the major research gaps in current literature lies in considering changes in phenology. Landsat-derived vegetation indexes such as Normalized Difference Vegetation Index (NDVI) usually represent a single summer day and are rarely calculated over different seasons or several years (e.g. Hystad et al. 2014; Klein et al. 2024). Similarly, street view imagery, which is increasingly used to map street greenery, is mostly collected in summer (Biljecki and Ito 2021; Hou et al. 2024). While the current practices inherently provide biased values on the availability of greenery (Klein et al. 2024), the significance of this bias is also poorly known. Little empirical research has examined whether street greenery during the leafless or driest time of the year can provide similar health and well-being benefits to other seasons, and how the magnitude of these changes might vary geographically. However, seasonal decline in active mobility has been shown, especially in countries with large seasonal changes (Frumkin et al. 2017; Kajosaari et al. 2022), which might be potentially attributed to changes in street vegetation.

**Fig. 1:**
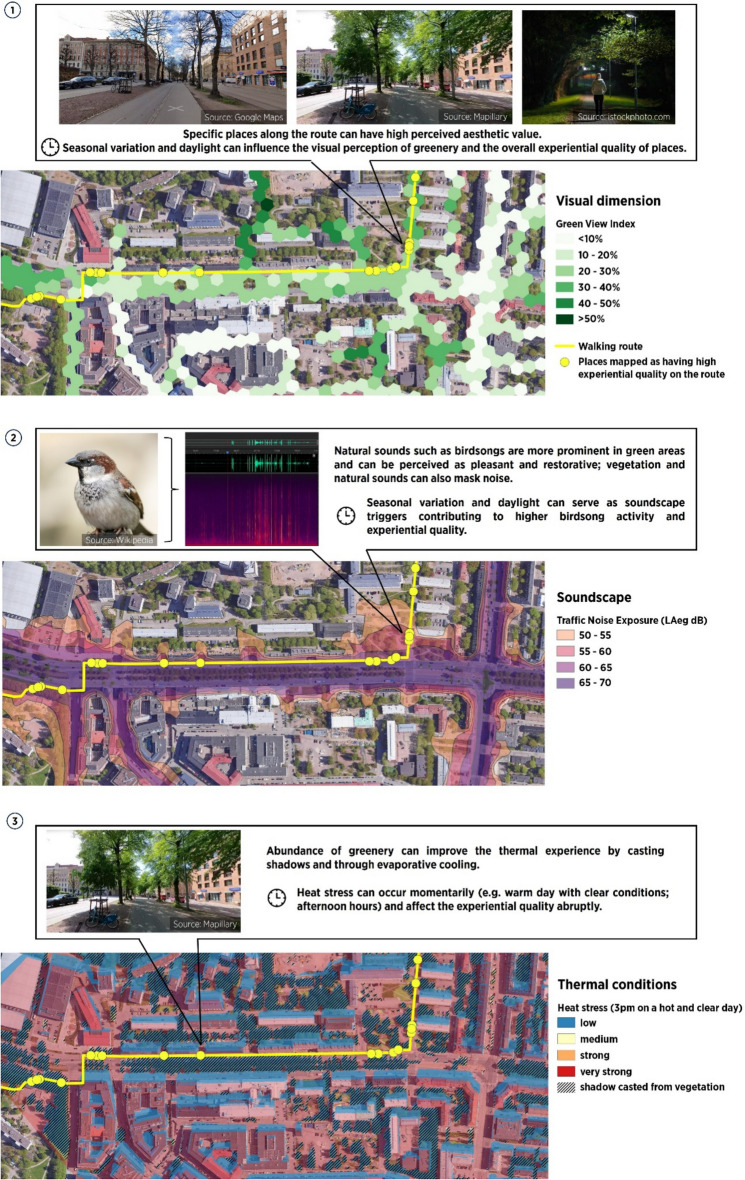
Integration of objective (GVI, noise, heat) and subjective (places of experiential quality) street greenery exposure assessments during walking on a street in Helsinki, Finland. Data and Methods: (1) Green View Index (GVI) obtained from Toikka et al. (2020); (2) Daytime road traffic noise 2022 (LAeq), retrieved from the Helsinki Map Service (City of Helsinki 2024); (3) Heat stress classifications according to Matzarakis et al. (1999). The underlying mean radiant temperature reflects heat conditions on 15 June 2023 and was modelled using the SOLWEIG tool (Lindberg et al. 2008). Input terrain models and land cover were retrieved from the Helsinki Map Service (City of Helsinki 2024) and meteorological parameters derived from Copernicus Climate Change Service (2019) and Copernicus Atmosphere Monitoring Service (2020). The walking route and places of high experimental quality en route are placed hypothetically for illustration

To illustrate these points, Fig. 1 presents a hypothetical example of street greenery exposure and experience while walking through a street in Helsinki, Finland. The figure combines multiple approaches for assessing the visual, acoustic and thermal dimensions, which serves two main purposes. First, it shows that there can be an important mismatch between objective and subjective assessments, especially due to differences in spatial scale and temporal dynamics. The objective assessments on the maps include eye-level greenery exposure measured through the Green View Index (GVI) (Toikka et al. 2020; Sánchez and Labib 2024), acoustic exposure measured through traffic noise levels (LAeq) (Babisch 2008; Bai et al. 2020) and thermal exposure measured through heat stress (Matzarakis et al. 1999; Lindberg et al. 2008). Such commonly used methods provide exposure averages that may fail to capture the diverse values and experiences of citizens. In this context, subjective assessments such as Public Participation GIS (PPGIS) surveys or Geographically-explicit Ecological Momentary Assessment (GEMA) mobile phone diaries can gather crucial location-based information on places of high or low experiential quality, and associated seasonal or daytime variations (yellow points in Fig. 1). This can include, for example, visual aesthetics of specific natural features such as large trees or seasonal flowers, soundscape affective quality (i.e. the perceptual and contextual attributes of the acoustic environment (Axelsson et al. 2010) or individual noise annoyance, and momentary heat stress. For instance, the traffic noise exposure data provides an average decibel (dB) level value indicating loudness, which reflects only one parameter of how humans perceive the acoustic environment. A person walking along a street can be exposed to birdsongs from park edges or street trees, or to vibrant sounds of human chatter, which may be positively associated with city life (Korpilo et al. 2023). In addition, Fig. 1 also exemplifies how using multiple exposure metrics simultaneously may yield different results. For instance, even if street trees are not sufficient to serve as a noise barrier, they can still provide shade, which can be crucial to a pleasant experience when cycling or walking during hot days (Tabatabaie et al. 2023). It is only through examining combined subjective and objective metrics that researchers and planners will get a clearer idea on what street greenery is important for what purposes, and how we can maximize multiple health-promoting and harm-reducing benefits.

## The WHERE: Where to increase or introduce street greenery?

Decisions on where to enhance or introduce street greenery for active mobility requires knowledge on current greenspace availability, accessibility, and mobility patterns. Technological and data advances such as high-resolution satellite images, street-view imagery, and AI models for automatic segmentation (Biljecki and Ito 2021; Labib et al. 2021), provide significant opportunities to measure spatial distribution patterns and identify potential lack of greenery. Similarly, mobility data and GPS tracking technologies can capture more accurately people’s spatio-temporal movements both at the micro-level of individual routes and macro-level of whole cities (Gariazzo et al. 2016; Poom et al. 2021). Such datasets can be combined with data on ecological connectivity, urban biodiversity, and spatial conservation to advise areas for prioritisation (Jalkanen et al. 2020). However, there is a clear need for context-specific, but comparable exposure assessment methods to support planners in locating street greenery where it serves multiple purposes. Previous studies have provided important recommendations in this context including the use of open-access finer resolution satellite images, selecting spatial units and scale of analyses based on the type of greenery exposure, and conducting inter-city and inter-country comparisons (Markevych et al. 2017; Labib et al. 2020).

Yet, such applications of methods should go hand in hand with inclusive planning and public engagement to support the prioritisation and design of urban street greenery. Related to our discussion in integrating objective and subjective assessments, Public Participation GIS methods can be a powerful tool in collecting diverse citizen insights, fostering inclusive planning, and providing valuable contextual information for planners (Kahila-Tani et al. 2019). Such surveys can involve citizens to comment on actual planning and development proposals or identify location-specific preferences for introducing new vegetation along travel routes. However, specific attention should be placed on issues of representation, which leads us to the last crucial point of discussion.

## The WHO: The role of street greenery in contributing to environmental (in)justice

Street greenery has been largely neglected in environmental and green justice literature (Wu et al. 2019). Overlooking greenery exposure and experience during everyday travel might provide a partial or even biased estimation of inequalities in the total daily exposure. For example, the availability of pleasant green travel routes may both compensate for a lack of residential greenery for some groups or, conversely, reinforce inequalities in greenery exposure for other groups (Wu et al. 2019; Wang et al. 2021). A few studies have shown that disadvantaged communities, such as low-income groups, often have less access and exposure to pleasant green travel environments (Łaszkiewicz and Sikorska 2020; Khanian et al. 2024). At the same time, there is some evidence that lower income groups can benefit more from public greenspaces as they tend to lack access to other health-promoting resources, which is referred to as the “equigenesis hypothesis” (Mitchell et al. 2015; Rigolon et al. 2021). Therefore, when designed properly, street greening interventions may benefit disadvantaged communities disproportionally, helping to narrow health-related inequalities. These findings highlight the importance of broadening the view on injustices to encompass the distribution of green travel environments and the consequent health and well-being (in)equalities these might bring.

Importantly, street greening in the urban space often entails trade-offs with other land use needs (e.g. new road infrastructure), which easily results into “winner” and “loser” streets with apparent implications on equity (Nello-Deakin 2024). This inherently links to another key consideration: who makes the decisions in transport greening interventions. Trade-offs between conflicting planning goals are imbedded in larger challenges in stakeholder collaboration. Previous research has identified various tensions and mismatches that can arise from differences in organisational and funding structures, planning goals and agendas, incompatible scales and timelines, group composition and power dynamics, planner’s individual characteristics, language and culture, and various nature values and knowledge systems (Berbés-Blázquez et al. 2016; Raymond et al. 2022; Branny et al. 2024). Recent studies have also highlighted that barriers in involving knowledge from citizens into urban spatial planning still exist, especially due to limited times and resources, as well as planners’ lack of experience, trust and willingness (Ramirez Aranda et al. 2023; Rossi et al. 2025). Nevertheless, research has also shown that different participatory processes can support the identification and reframing of tensions through reflexivity, communication, and building of partnerships, trust and empathy across actors, ultimately contributing to more inclusive greening interventions (Lawrence et al. 2022; Raymond et al. 2022).

## Conclusion and summary of key research priorities

European cities are on an ambitious roadmap to achieve new nature restoration goals. There is a great potential for street greenery to act as a key player in increasing overall urban greenspace and tree canopy cover even in dense urban areas, while simultaneously enhancing sustainable mobility, human health and well-being, and reducing environmental injustice. However, planners and practitioners responsible for urban greening need more evidence. Therefore, we call for future research to prioritize several directions:

**Understanding the underlying mechanisms of street greenery-health interactions in the context of moving through space.** This requires examining the unintentional, rapid, and momentary exposure during everyday active mobility among different groups and geographical contexts. Here, longitudinal, intervention and experimental studies play a vital role.

**Use of multiple exposure measures with attention to spatial and temporal dynamics** to study what types, quantity and quality of street greenery are important for well-being and health benefits during active mobility. Approaches should be context- and exposure-specific, and sensitive to spatial (e.g. high-resolution images, adjusted buffers) and temporal scales (e.g. seasonally-sensitive green indices). At the same time, methods for analysing local spatial variation between exposure metrics and health should be sufficiently standardized (e.g. by using Geographically Weighted Regression models).

**Integration of objective and subjective street greenery assessments** to measure not only the presence and absence of greenery, but also realized mobility, accessibility, and greenspace values and preferences of different urban populations. This requires the integration of active (e.g. Public Participation GIS surveys) and passive (e.g. satellite, street view imagery and mobile Big Data) sensing methodologies to untangle not only differences between but also within different social-economic groups. For example, surveys can help to uncover the potential equigenic effect of street greening interventions for low-income communities.

**Focus on inclusive planning that accounts for synergies and trade-offs:** Paying more attention to street greenery is also crucial for a more inclusive urban planning. Nowadays, it is highly unlikely that large green spaces can be added or built up in dense urban areas, thus, planting street greenery can provide a more feasible solution to reduce environmental injustices and achieve overall sustainability goals.

Finally, it is important to acknowledge that greening interventions lead to trade-offs not only between social and ecological goals, but also within human and other species groups. There is limited space in cities and building new active mobility infrastructure may compete with space for adding greenery and vice versa. Therefore, it is crucial to find synergies between multiple functions and those who benefit, so that restoration outcomes can be maximized.
